# Retraction: Physiological basis and image processing in functional magnetic resonance imaging: neuronal and motor activity in brain

**DOI:** 10.1186/1475-925X-12-113

**Published:** 2013-10-18

**Authors:** Rakesh Sharma, Avdhesh Sharma

**Affiliations:** 1 Departments of Medicine and Radiology, Columbia University, New York, NY 10032, USA; 2 Electrical Engineering, Indian Institute of Technology, New Delhi, 110016, India

## Retraction

This article [1] has been retracted by the publisher because of significant overlap with figures from previously published work [2,3] without appropriate attribution or permissions. We were unable to contact the authors of the article, despite our best efforts. We apologise to all affected parties for the inconvenience caused.
